# Editorial: Neonatal health in low- and middle-income countries. Now is the time

**DOI:** 10.3389/fped.2023.1168915

**Published:** 2023-04-13

**Authors:** Andrew P. Steenhoff, Susan E. Coffin, Ashish KC, Britt Nakstad

**Affiliations:** ^1^ Global Health Center & Division of Infectious Diseases, The Children’s Hospital of Philadelphia, Philadelphia, PA, United States; ^2^ Department of Pediatrics, University of Pennsylvania, Philadelphia, PA, United States; ^3^ The Botswana-UPenn Partnership, Gaborone, Botswana; ^4^ Department of Paediatric & Adolescent Health, Faculty of Medicine, University of Botswana, Gaborone, Botswana; ^5^ School of Public Health and Community Medicine, Sahlgrenska Academy, University of Gothenburg, Gothenburg, Sweden; ^6^ Department of Women’s and Children’s Health, Uppsala University, Uppsala, Sweden; ^7^ Division of Paediatric and Adolescent Medicine, Institute of Clinical Medicine, University of Oslo, Oslo, Norway

**Keywords:** neonatology, LMIC, newborn, neonatal mortality, low resourced countries

**Editorial on the Research Topic** Neonatal health in low- and middle-income countries. Now is the time

The first 28 days after birth—the neonatal period—is the most vulnerable month for a child (1). The global “Every Newborn” action plan, which was endorsed by the WHO General Assembly in 2014, sets out an ambitious vision of a world with no preventable stillbirths or neonatal deaths (2). The action plan, endorsed in 2014 by 194 member states, articulates a goal that all countries reach the target neonatal mortality rate (NMR) of 12 or less newborn deaths per 1,000 live births by 2030, as well as commit to ongoing work to reduce death and disability. Achieving this interim goal is essential to ensure that no newborn is left behind. This metric was adopted as a target for Sustainable Development Goal (SDG) Target 3.2 (3). Despite a decline of more than 50% in the global neonatal mortality between 2000 and 2021, 2.4 million neonatal deaths still occur each year. The majority (79%) of these deaths take place in countries in South Asia and Sub-Saharan Africa, where the social system and health care settings are inadequately resourced.

This editorial team was pleased to use this special issue as an opportunity to identify new frontiers in global neonatal health, especially in the context of two global crises—the SARS-CoV-2 pandemic and climate change. We received 65 manuscripts, of which 40 (62%) were accepted. These 40 included 27 (68%) original research manuscripts, five (13%) brief research reports, three (8%) perspective pieces, two (5%) reviews, one (3%) case report, one (3%) on hypothesis and theory, and one (3%) on methods. First authors represented 15 countries; six in Africa (Botswana, Ethiopia, Nigeria, South Africa, Tanzania, Uganda), five in Asia (Bangladesh, China, India, Japan, Malaysia), three from Europe (Sweden, Switzerland, United Kingdom) and one in North America (United States). Of the 40 accepted papers the countries with the most first authors were South Africa (8; 20%), United States (7; 18%) and Ethiopia (5; 13%). Issues related to suboptimal study design or biostatistical analysis led to the rejection of 25 submitted papers.

Multiple factors influence neonatal health in LMIC, so a broad range of topics were included. High-risk preterm neonates in China were followed more effectively in a family-centred, child-friendly multidisciplinary clinic, leading to an earlier diagnosis of neurodevelopmental impairment and cerebral palsy (Huang et al.). In Ethiopia, less than 10% of neonates received post-natal check-ups. Increasing antenatal care (ANC) visit utilization, improving institutional delivery, raising awareness about neonatal danger signs, increasing access to health care facilities, and implementing home-based neonatal care visits by healthcare providers could all help to improve postnatal check-ups (Birhane et al.).

The Every Mother Every Newborn (EMEN) tools can reasonably measure WHO/UNICEF/UNFPA quality standards (Siseho et al.). In India, the quality and impact of home visitation services remains challenging and can be enhanced by addressing the social-cultural, organizational, educational, economic, and physical nexus domains with concurrent efforts for skill and confidence enhancement of the Accredited Social Health Activists (ASHAs) and their credibility (4).

Most mothers in rural Ethiopia had limited knowledge of Early Newborn Care (ENC). There is opportunity to enhance maternal knowledge pertaining to cord care, breastfeeding, and thermal care by improving access to ANC and institutional delivery (Siseho et al.). In Bangladesh there is room for improvement of the entire maternal care continuum—from ANC to facility delivery and postnatal care (PNC) to improve care-seeking for the sick newborn. Strategies such as referral training for unqualified providers, targeted intervention for poorer households, increasing community health care worker (CHW) home visits and neonatal danger sign counselling at the facility and community could address these issues in many LMIC (Getachew et al.; Azad et al.).

A number of papers explored aspects of Kangaroo Mother Care (KMC). In a neonatal cohort from South Africa, both HIV-exposed uninfected and HIV unexposed uninfected groups of infants showed reasonable weight gain regardless of maternal HIV status (Getachew et al.). In Uganda implementation of KMC has been suboptimal, despite wide acceptance (Azad et al.), highlighting post-discharge challenges in rural and resource-limited settings. The study provided insights on KMC implementation and sustainability from the perspectives of key stakeholders, highlighting the need for a holistic approach to KMC that incorporates its adaptability to community settings and contexts. Gambian researchers demonstrated the impact of health system limitations on successful delivery of KMC, and suggested that linkage to comprehensive health care worker (HCW) and KMC provider education would enhance effectiveness, safe delivery and monitoring (Mapatha et al.). Further context specific research into safe and respectful implementation is required from varied settings and should include perceptions of all stakeholders, especially if there is a shift in global policy toward KMC for all small vulnerable newborns (Mapatha et al.; Kwesiga et al.; Cho et al.).

As LMIC health systems continue to advance, the complexity of care also increases. One example is the correction of complex birth defects such as bladder exstrophy (BE). BE requires a complex, long-term course of care and in India this significantly impacted caregiver distress (Spencer et al.). Mental health screening for caregivers of children with complex congenital anomalies like BE, should be an essential element of any comprehensive effort to alleviate the global burden on mothers and families of neonates born with congenital anomalies.

Newborn nutrition is crucial. Feeding practices for very preterm and VLBW infants vary widely within Nigeria and Kenya, likely because of lack of locally generated evidence. High quality research that informs the feeding of these infants in the context of limited human resources, technology and consumables is urgently needed (Tongo et al.). Vitamin insufficiency should be checked (Wang et al.) as well as awareness of maternal undernutrition (Bilal et al.) and poor neonatal care in conflict settings (Kampalath et al.).

Health systems strengthening at all levels and quality improvement work is needed to improve global neonatal health and reduce death rates. Factors influencing neonatal mortality, morbidity and outcomes (Ramdin et al.; Ingemyr et al.; Shiferaw et al.; Mokuolu et al.; Adugna and Worku; McCulloch et al.) were discussed in several papers, especially preterm babies (Mangiza et al.; Hailemeskel and Tiruneh) as well as a need to optimize treatment of respiratory distress syndrome with appropriate and affordable non-invasive equipment (Ekhaguere et al.). Several studies examined factors associated with poor neonatal outcome, emphasizing that the quality of newborn care around the time of birth and prior to discharge is essential. In South Africa's largest hospital almost one-third of babies born by Caesarian section develop moderate (27%) or severe (2%) hypothermia (Siseho et al.; Patel et al.). Poor outcomes may also follow discharge against medical advice (DAMA). In China, the rate of DAMA in preterm infants remains high (14%) with a significant impact on neonatal mortality rates (Xiu et al.). Continuous efforts to reduce hypothermia after Caesarian delivery and minimize neonatal DAMA would likely result in substantial improvement of outcomes for infants in many parts of the world. It is essential to have available locally-appropriate technologies to avoid hypothermia after birth (5) as well as enable effective screening of neonatal jaundice (Suzuki et al.). To reduce risk of complications of neonatal hypoxic ischemic encephalopathy in LMIC, hypothermia treatment is warranted but challenging to perform [Birhane et al. (5), Boo et al.]. Other papers emphasized the need for newborn resuscitation training with video-recording (Olson et al.), and that intramuscular epinephrine in a neonatal animal model (Berkelhamer et al.) was not effective. Training of NICU staff is important to strengthen basic neonatal care practises (Swanson et al.), not least when the ward is understaffed and overcrowded. Complications of prematurity as well as congenital conditions and options for adequate treatment in LMIC were discussed in various papers (Spencer et al.; Xiu et al.; Kesting and Nakwa; Vidavalur; Liu et al.). Last, but not least, several papers focused on neonatal infection prevention, antibiotic stewardship, antibiotic resistance patterns (Johnson et al.; Holgate et al.; Shah et al.), and infectious diseases that are more common in warm climate LMICs (Mapatha et al.; Nakstad et al.; Nakwa et al.; Sundararaman and Odom John).

We wish to highlight ongoing challenges of achieving scientific equity for work being done in LMIC. Ensuring that physicians, scientists, and policy makers from all parts of the globe have easy access to these publications was essential to our editorial team. While open access publications achieve this “access” goal, we acknowledge that publication fees represent a barrier to many scientific authors. This barrier disproportionately affects authors from LMICs who have less access to research funding. This in turn perpetuates a systemic publication bias in favour of data from high-income countries (HIC) and stunts science in LMICs. We encourage journals to find the sweet spot of open access, affordability for authors and journal financial sustainability. An excellent example of how this might be accomplished is the recently launched Journal of African Neonatology (6). This biannual peer-reviewed open-access journal published by the African Neonatology Association accepts and publishes manuscripts in English and French with a publication fee of US$50. Indeed, now is the time for major progress in neonatal health in LMIC.

We also acknowledge that this research topic only accepted manuscripts in English favouring some authors but placing many at a linguistic disadvantage. A number of papers benefitted from detailed copy editing by generous, dedicated reviewers and we acknowledge and sincerely thank them for their contributions towards scientific equity.

This collection of articles is timely, inspiring, informative, and crucial, but given the grand scope and pressing nature of the topic, incomplete. With an eye to the future, we hope that this collection will be part of a “publication shift” where many future research topics will be dedicated to neonatology in LMIC and that journals will increasingly focus on highlighting scholarship to advance neonatal care in LMIC. Guided by expert opinion and evidence for equitable partnerships in global child health [Steenhoff et al. (7)], let us each play our role in advancing the care of neonates in LMIC.
